# Lipoma Arborescens in the Knee Joint

**DOI:** 10.7759/cureus.89391

**Published:** 2025-08-05

**Authors:** Vishwas Kadambila, Vishakh Karicheri Gangadharan, KS Arif, Sudharshan Abhishek BS

**Affiliations:** 1 Orthopaedics, Kanachur Institute of Medical Sciences, Mangalore, IND

**Keywords:** arthroscopic synovectomy, frond-like synovial mass, knee swelling, lipoma arborescens, rare knee tumor

## Abstract

Lipoma arborescens (LA) is an unusual and benign lesion found in joints, where one can see villous lipomatous synovial proliferation, with the knee joint being the commonest site. It typically presents with chronic joint swelling and pain.

We report the case of a 32-year-old woman with a history of knee trauma three years ago, who presented with progressive pain and swelling in the left knee. Examination revealed swelling with a boggy consistency over the suprapatellar area. There was no joint line tenderness, and the routine clinical tests did not reveal any abnormality. Magnetic resonance imaging indicated synovial proliferation with frond-like synovial mass in the suprapatellar pouch, lateral aspect. Synovial debridement was planned after, which was then performed arthroscopically. Synovial biopsy histopathological examination confirmed the diagnosis of lipoma arborescens. Postoperatively, the patient recovered uneventfully and was able to pursue daily activities without any hindrance.

Lipoma arborescens is a rare condition and should be considered in patients with chronic knee swelling and clinical and radiological features suggestive of florid synovitis. Arthroscopic debridement remains an effective treatment.

## Introduction

Lipoma arborescens (LA) is a relatively rare clinical condition first described by Hoffa in 1904 [1]. It is characterized by diffuse villous proliferation of the synovium with mature fat cell infiltration. Although the knee joint is the most commonly affected site, it can also occur in other joints such as the hip, shoulder, and elbow. The condition typically presents in adults between the fifth and seventh decades of life and is associated with chronic joint swelling and intermittent pain. Because of its low incidence, lipoma arborescens is usually missed by clinicians as it can often mimic other common synovial and joint pathologies, which can lead to treatment being delayed [2]. The etiology is often idiopathic but can be secondary to trauma or chronic irritation. At present, the number of reports on this condition and its treatment using arthroscopy is minimal.

## Case presentation

A 32-year-old woman presented to our outpatient department with complaints of intermittent left knee progressive pain for the past three months. She had a history of a slip and fall three years back, resulting in an injury to her left knee that was managed conservatively at a local hospital, and the pain subsided over time. Examination revealed swelling with a boggy consistency over the suprapatellar area. There was no joint line tenderness, and the clinical tests for cruciates, collaterals, and menisci were negative. Range of motion was full. Neurovascular examination was normal. Infection parameters in the blood, including C-reactive protein (CRP), erythrocyte sedimentation rate (ESR), and total and differential leucocyte counts, were within normal limits.

**Figure 1:**
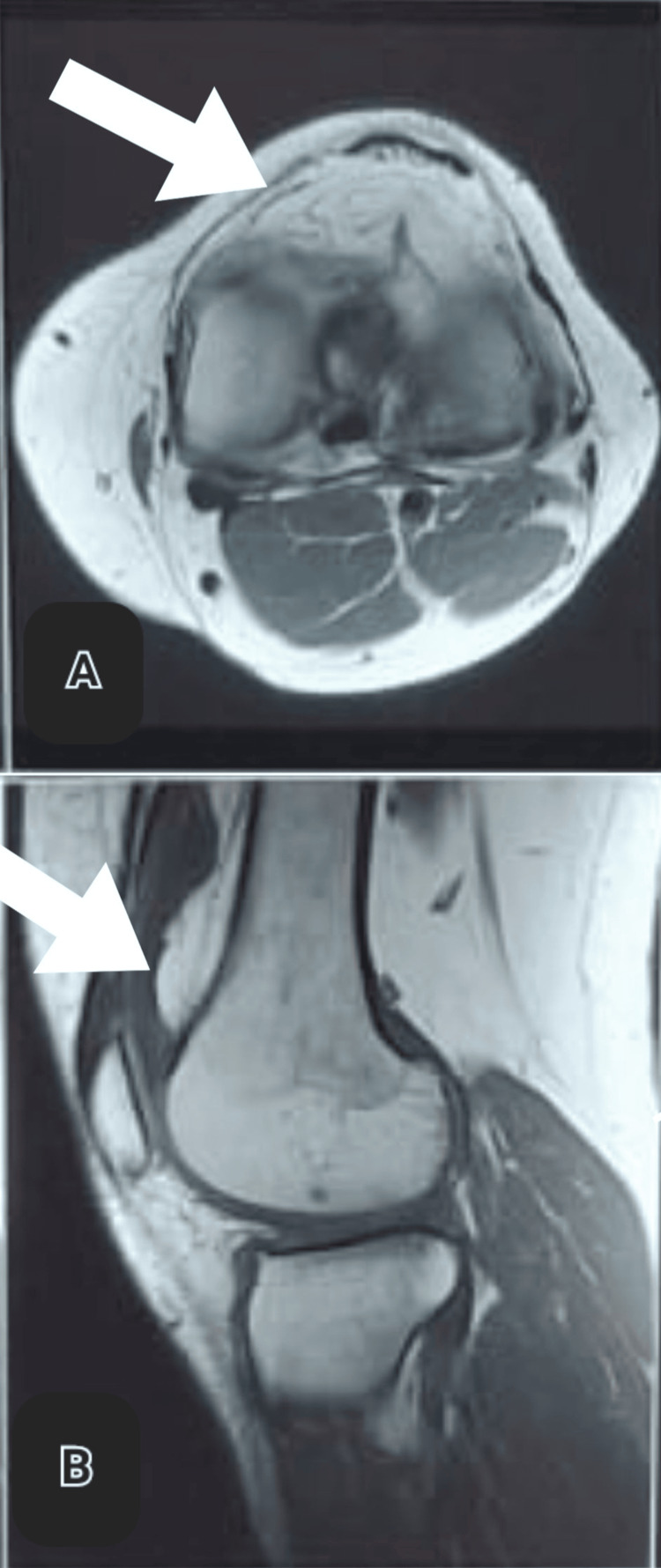
MRI images of the pathology A: axial image, B: sagittal image Arrows indicate the frond-like arborescent synovial mass. MRI: magnetic resonance imaging

Magnetic resonance imaging indicated synovial proliferation with frond-like synovial mass measuring 31 × 11 × 21 mm in the suprapatellar pouch, lateral aspect (Figure 1).

**Figure 2:**
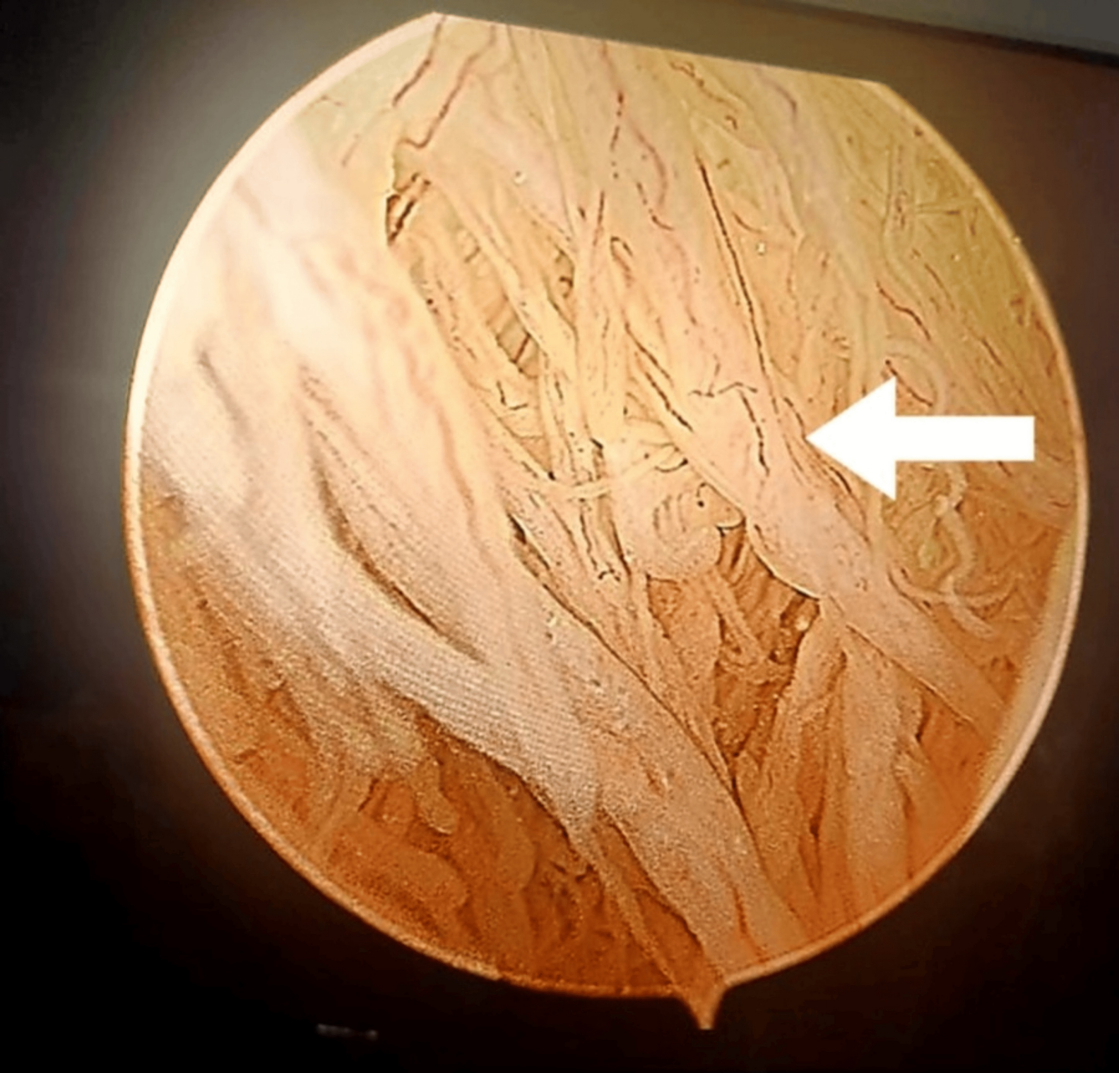
Arthroscopic image of the pathology The arrow indicates frond-like fatty villous synovial proliferations.

On arthroscopy, extensive synovial villous proliferation was visualized (Figure 2).

As a matter of fact, it looked like we were in the bowel (with all the villi) rather than the knee (Video 1).

**Video 1:** Arthroscopic video of the pathology

Radical synovial debridement was performed, and samples were sent for biopsy. Histopathological analysis confirmed the diagnosis of lipoma arborescens.

Postoperatively, the patient had significant symptom relief and regained normal knee function. At one-year follow-up, there was no recurrence, and she reported no difficulty performing her daily activities.

## Discussion

Lipoma arborescens is a rare and usually missed cause of chronic knee swelling and pain. Fewer than 200 cases have been reported in the literature, with the knee joint being the most common site. It is a synovial disorder whose etiology is not known. Synovial villous lipomatous proliferation is its hallmark. Lipoma arborescens usually presents between the ages of 50 and 70 years, with equal incidence in both men and women [3]. The lesion is seen in the intra-articular region, grows very slowly, has a chronic pattern, and is benign. The suprapatellar recess of the knee joint is the classic site, with less commonly reported sites being the hip, shoulder, and elbow. It is usually unilateral, but multiple and bilateral involvement has also been documented [4].

The exact etiology remains unclear. Some authors suggest a reactive process secondary to chronic irritation, trauma, or inflammatory arthropathies. Several case reports highlight the association between prior trauma and LA development [5]. Patients usually present with chronic, painless swelling. Pain, when present, is often due to secondary synovitis or mechanical irritation. The hypertrophied adipose-laden synovial villi can get entrapped between the joint surfaces and can worsen the clinical features. Routine blood tests tend to be normal. Synovial fluid cultures show no growth, and the aspirate has no cells or crystals [6]. Precise identification of lipoma arborescens requires a robust clinical examination with suspicion of the same in mind.

Proliferation of adipose tissue in the synovial cavity is identified by magnetic resonance imaging (MRI). The typical MRI features of lipoma arborescens in the knee joint include a synovial mass with a frond-like arborescent structure in the suprapatellar femoral area. This mass lights up like fat across all pulse sequences, but with a suppressed signal in fat saturation/short TI inversion recovery (STIR) sequences. Hypertrophied synovium in T1 gives an intermediate signal and a high signal in T2 or STIR. Joint effusion, if present, can also be seen [7]. Additionally, degenerative changes in the joint and tears of the meniscus can be visualized, which can act as a contributing factor to the development of lipoma arborescens.

The diseases that can mimic lipoma arborescens include synovial chondromatosis, pigmented villonodular synovitis (PVNS), hemangioma of synovium, and rheumatoid arthritis (RA). The definitive diagnosis can be made by histopathological examination [8]. Microscopic analysis reveals mature adipocytes infiltrating the synovium, a characteristic feature that differentiates it from other pathologies affecting the joint.

The treatment is open synovectomy or arthroscopic synovectomy, where the proliferative adipose tissue is removed to restore the function of the joint [9]. Arthroscopic synovectomy provides a minimally invasive treatment modality, facilitating early and faster recovery as compared to conventional open surgery. After surgery, recurrence is minimal, hence providing good outcomes.

## Conclusions

This case emphasizes the importance of considering lipoma arborescens in patients with persistent knee swelling, especially those with a history of trauma. A synovial mass with a frond-like arborescent structure can be seen in MRI, and a synovial biopsy helps clinch the diagnosis, where one can visualize mature adipocytes infiltrating the synovium. Arthroscopic synovial debridement remains an effective treatment, providing symptomatic relief and functional recovery.
